# Molecular sled is an eleven-amino acid vehicle facilitating biochemical interactions via sliding components along DNA

**DOI:** 10.1038/ncomms10202

**Published:** 2016-02-02

**Authors:** Walter F. Mangel, William J. McGrath, Kan Xiong, Vito Graziano, Paul C. Blainey

**Affiliations:** 1 Department of Biology, Brookhaven National Laboratory, 50 Bell Avenue, Upton, New York 11973, USA; 2 Department of Biological Engineering, Massachusetts Institute of Technology, Cambridge, Massachusetts 02139, USA; 3 Broad Institute of MIT and Harvard, Cambridge, Massachusetts 02142, USA

## Abstract

Recently, we showed the adenovirus proteinase interacts productively with its protein substrates *in vitro* and *in vivo* in nascent virus particles via one-dimensional diffusion along the viral DNA. The mechanism by which this occurs has heretofore been unknown. We show sliding of these proteins along DNA occurs on a new vehicle in molecular biology, a ‘molecular sled' named pVIc. This 11-amino acid viral peptide binds to DNA independent of sequence. pVIc slides on DNA, exhibiting the fastest one-dimensional diffusion constant, 26±1.8 × 10^6^ (bp)^2^ s^−1^. pVIc is a ‘molecular sled,' because it can slide heterologous cargos along DNA, for example, a streptavidin tetramer. Similar peptides, for example, from the C terminus of β-actin or NLSIII of the p53 protein, slide along DNA. Characteristics of the ‘molecular sled' in its milieu (virion, nucleus) have implications for how proteins in the nucleus of cells interact and imply a new form of biochemistry, one-dimensional biochemistry.

## Abstract (web summary)

Extremely compact environments can inhibit protein interactions by preventing 3-dimensional diffusion. Here the authors show that an 11-amino acid long peptide can function as a ‘molecular sled', able to transport cargo linearly along DNA to enable interactions.

## Graphical abstract

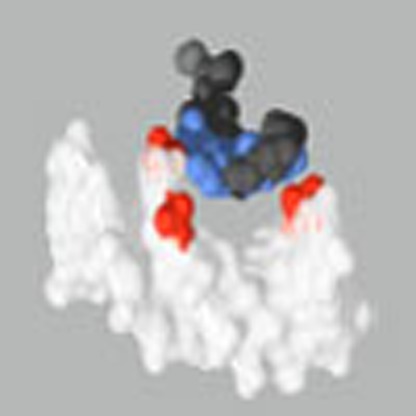

The adenovirus proteinase (AVP), a 23 kDa cysteine proteinase^1^,^2^, is activated inside a nascent virion, so it can cleave multiple copies of virion precursor proteins used in virion assembly^3^,^4^. Only then is the virus particle infectious. AVP is activated by two cofactors, pVIc, the 11-amino acid peptide (GVQSLKRRRCF) from the C terminus of the precursor to adenovirus protein VI, pVI, and the double-stranded linear viral DNA genome of about 35,000 bp (refs 5, 6). The cofactors increase the k_cat_/K_m_ for substrate hydrolysis^7^,^8^,^9^,^10^. In the presence of DNA, the activity of AVP increases 11-fold; in the presence of pVIc it increases 118-fold^7^. In the presence of both cofactors, the activity of AVP increases synergistically, 34,000-fold. Inside nascent virus particles, the activated AVP and its protein substrate pVI slide on DNA over hundreds to thousands of base pairs, depending in part on the ionic strength, via one-dimensional diffusion to facilitate productive interactions with DNA-bound precursor protein substrates. Facilitation is needed, because the high concentration of viral DNA prevents these sequence-independent DNA-binding proteins from diffusing effectively in three-dimensional space as they are essentially irreversibly bound to the viral DNA^5^,^10^.

AVP, which is synthesized in an inactive form, is partially activated by pVI by forming AVP–pVIc complexes according to the following mechanism: AVP binds randomly to DNA and does not slide^11^. pVI also binds randomly to DNA, but it slides along DNA with a significant one-dimensional diffusion constant, 1.45 × 10^6^ (bp)^2^ s^−1^ (ref. 12). pVI slides into AVP. AVP, partially activated by being bound to the viral DNA, cleaves pVI first at its N terminus and then at its C terminus. pVIc, released by cleavage of pVI at its C terminus, binds to the AVP molecule that cut it out^11^. A disulfide bond is formed between Cys10′ of pVIc and Cys104 of AVP resulting in the very active AVP–pVIc complex, an irreversibly activated form of the enzyme. The processing of the virion proteins, cleavage at >2,000 sites per virion by ∼50 AVP–pVIc complexes per virion^13^, occurs by the following mechanism: covalent, active AVP–pVIc complexes bound to DNA, the most active form of the enzyme, slide along the viral DNA with a one-dimensional diffusion constant of 21.0 × 10^6^ (bp)^2^ s^−1^ to encounter and cleave precursor proteins, which are also nonspecifically bound to the viral DNA^14^.

Here we ask by what mechanism do pVI and AVP–pVIc complexes slide along DNA to render virus particles infectious? We show that the 11-amino acid peptide pVIc is necessary and sufficient for sliding. pVIc alone binds to DNA independent of DNA sequence and slides along DNA. Furthermore, pVIc is a ‘molecular sled,' because it not only slides AVP and protein VI along DNA, it can also slide heterologous cargos. For example, streptavidin tetramers do not bind to DNA. Streptavidin tetramers attached to pVIc not only bind to DNA but also slide along DNA. Fragments of human proteins homologous to pVIc, the 8 amino acids at the C terminus of β-actin and a 13-amino acid peptide containing nuclear location signal III, NLSIII, of the p53 protein, exhibit similar DNA binding and sliding activities. This suggests the DNA contour may be a previously unrecognized venue for productive protein–protein interactions via one-dimensional biochemistry.

## Results

### A role for pVlc in sliding along DNA

By what physical mechanism do pVI and AVP–pVIc complexes slide along DNA to render nascent adenovirus virus particles infectious (Supplementary Discussion)? Both proteins have in common pVIc, whether as the C terminus of pVI or as the 11-amino acid peptide cofactor covalently attached to AVP in the AVP–pVIc complex. Whereas AVP–pVIc complexes slide on DNA^14^, AVP alone does not slide^11^, although it binds to DNA^10^. This raises the possibility that pVIc is a modular component able to confer sliding ability on proteins to which it is attached, for example, AVP and protein VI. If so, does its binding induce conformational changes that lead to the appearance of a sliding interface able to interact with DNA or does pVIc itself present such a sliding interface?

### pVIc binds to DNA

**Figure 1:**
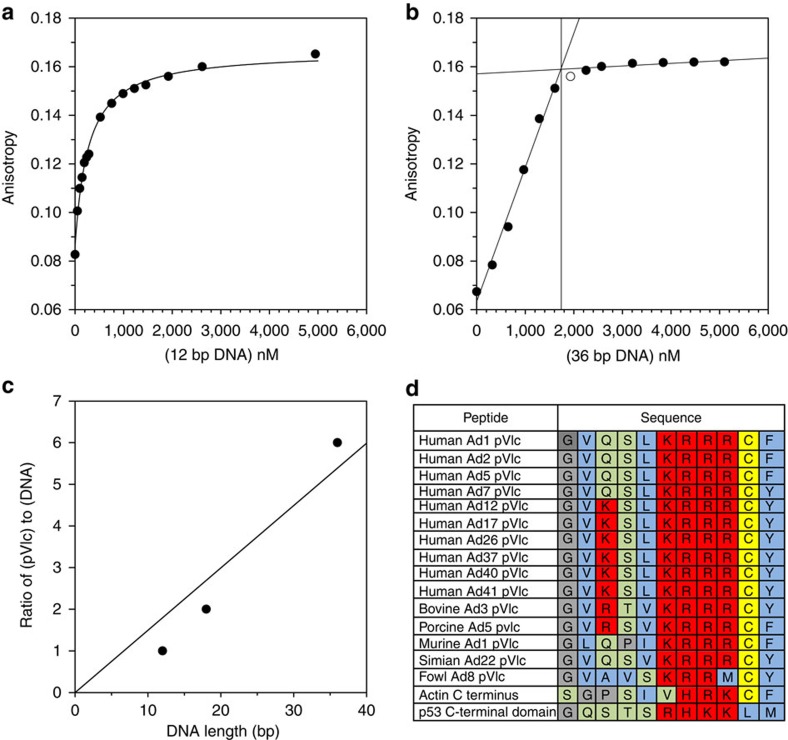
Binding of pVIc to DNA. (**a**) The equilibrium dissociation constant, *K*_d(app.)_ for the binding of pVIc to DNA was determined by incubating 10 nM Cy3B-pVIc with increasing amounts of 12-mer dsDNA and measuring the change in anisotropy. The experiment was repeated five times. The line through the closed circles represents the nonlinear regression fit of the experimental data to a 1:1 ligand–receptor model. The *K*_d(app.)_ was 264±25 nM (ref. 14). (**b**) The stoichiometry of binding of pVIc to DNA was determined by incubating 10 nM Cy3B-pVIc and 10 μM pVIc with increasing amounts of 36-mer dsDNA and measuring the change in anisotropy. The two straight lines were drawn using the data in the filled-in circles. The point with the open circle was not included in the fits for the lines. The intersection point of the two lines is the minimal concentration of DNA required to saturate 10 μM pVIc; it indicates six molecules of pVIc saturate one molecule of 36-mer dsDNA. (**c**) The number of base pairs of DNA occluded by the binding of one pVIc to DNA. The experiment in **b** was repeated but with 12-mer and with 18-mer dsDNA, and the stoichiometries of binding plotted versus the DNA length in base pairs. The line indicates that one molecule of pVIc occupies 6.6 bp of dsDNA. (**d**) Amino-acid sequences of pVIcs from various adenoviruses, the last eight amino acids of β-actin and NLSIII from the p53 protein. Basic amino acids are coloured in bright red, hydrophobic in blue, polar in light green, cysteine in light yellow, and glycine and proline in light grey.

If pVIc is a modular component able to confer sliding ability on proteins, then, it may be able to bind to DNA by itself and slide on DNA by itself (Supplementary Note 1). pVIc binds to DNA (Fig. 1a) with a *K*_d(app.)_ of 264±25 nM (Table 1). Six to seven base pairs of DNA are occluded (Supplementary Note 2) by the binding of one pVIc molecule to DNA (Fig. 1b,c and Table 1). Because the number of pVIc molecules that can bind to DNA is proportional to the length of the DNA, we conclude that binding is independent of DNA sequence (Fig. 1b,c). The amino-acid sequence of pVIc is conserved, Fig. 1d. The crystal structure of pVIc in the AVP–pVIc complex^1^,^2^ is highlighted in Supplementary Fig. 1; it shows that the pVIc moiety in the complex is exposed to solvent and, therefore, capable of interacting with DNA.

### pVIc slides on DNA

**Figure 2:**
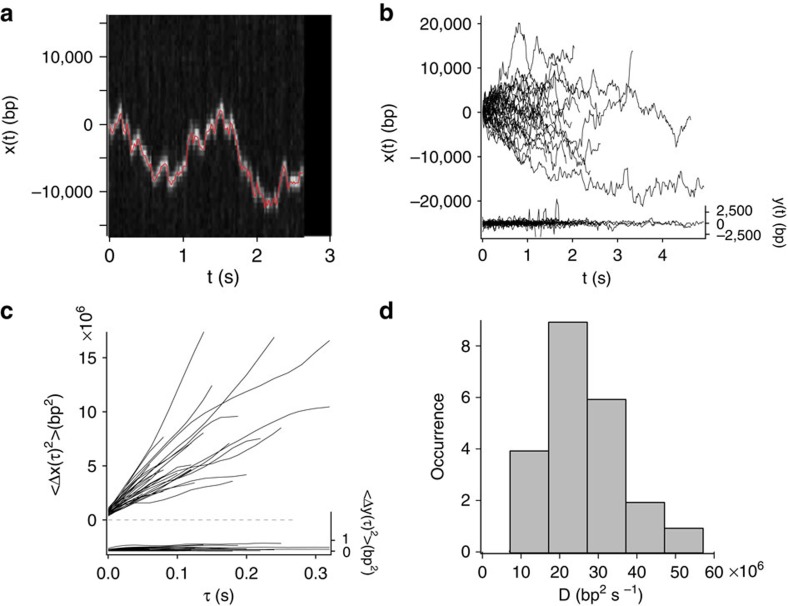
One-dimensional diffusion of pVIc along DNA. (**a**) Rapid motion of a pVIc molecule along flow-stretched dsDNA recorded at 50 frames per second. The kymograph was generated from raw images showing motion along DNA (each vertical strip of pixels is taken from one movie frame) as a function of time (horizontal axis). The red line represents the position estimate in each frame determined using Gaussian centroid determination. (**b**) Diffusion of pVIc along flow-stretched dsDNA. The 11-amino acid peptide pVIc diffuses rapidly along DNA (*x*(*t*), left axis, 35 trajectories). (**c**) MSD of the trajectories shown in **b** along the DNA (<Δ*x*(τ)^2^>, left axis). (**d**) Histogram of the diffusion constants for pVIc diffusing along dsDNA. The initial slopes of the MSD of each of the 35 trajectories from pVIc molecules sliding on DNA plotted in **c** were used to calculate one-dimensional diffusion constants (*D*_1_) according to *D*_1_=<Δx^2^>/2Δτ. The results are displayed in the histogram; the mean was equal to 26.0 × 10^6^ (bp)^2^ s^−1^ (ref. 14). In **b** and **c** motion transverse to the DNA (*y*(*t*) and *<*Δ*y*(τ)^2^>, respectively, right axes) is represented on the same scale, as a control.

To see if pVIc slides on DNA, we performed a facilitated diffusion assay (Supplementary Discussion) using total internal reflection fluorescence microscopy with flow-stretched dsDNA at pH 6.5 in 2 mM NaCl and pVIc labelled with a single fluorescent dye molecule^15^,^16^. Individual pVIc molecules were observed not only binding to random locations on the DNA as predicted^5^,^10^, but, surprisingly, they were observed diffusing along the DNA as well. Indeed, pVIc exhibited robust sliding activity, with many molecules remaining bound to the DNA for longer than a second, some up to 6 s, while sliding over tens of thousands of base pairs (more than half the length of the adenovirus genome). For example, the molecule whose motion is shown in the raw image data in Fig. 2a travelled over >30,000 base pairs during a 3 second binding event. Despite the optical resolution limit imposed by diffraction, the centroid position (see Methods section) of each signal in each frame was determined with sub-diffraction-limited resolution, <50 nm (red line in Fig. 2a).

The one-dimensional diffusion constant of pVIc was the highest average value yet reported for diffusion along DNA. The trajectories of 35 pVIc molecules sliding randomly in both directions on DNA are plotted in Fig. 2b. The mean square displacement (MSD) of each molecular trajectory shown in Fig. 2b is plotted in Fig. 2c. The MSD for each molecule was approximately linear with diffusion time, indicating transport dominated by Brownian motion. From the MSD initial slopes, one-dimensional diffusion constants (*D*_1_) were estimated according to *D*_1_=<Δ*x*^2^>/(2Δτ) and are displayed in the histogram in Fig. 2d. The mean one-dimensional diffusion constant was 26±1.8 × 10^6^ (bp)^2^ s^−1^ with an s.d. of 11 × 10^6^ (bp)^2^ s^−1^ (Table 1).

Given this large one-dimensional diffusion constant, we considered the possibility that processes other than sliding along DNA might be occurring (Supplementary Note 3)^17^. Sliding consists of one-dimensional translocation along the DNA with the polypeptide in continuous contact with the DNA^16^,^17^,^18^. Hopping can be mistaken for sliding in tracking experiments. Hopping occurs when the polypeptide repeatedly dissociates from the DNA and rebinds at nearby locations on the DNA, in a microdiffusion process^19^,^20^. If hopping were occurring, the diffusion constant should increase as the salt concentration is increased, since the residence time on DNA decreases. Experiments at a 10-fold-higher salt concentration, 20 mM, showed the one-dimensional diffusion constant to be slightly lower, 17.9±3.5 × 10^6^ (bp)^2^ s^−1^ with an s.d. of 10.7 × 10^6^ (bp)^2^ s^−1^ (Supplementary Fig. 2 and Table 1), thereby ruling out hopping as the primary means by which pVIc moves along the DNA in these experiments.

### Sliding of Adenovirus proteins along DNA mediated by pVIc

pVIc probably contributes the majority or the entirety of the sliding contacts, contacts between protein and DNA in all the contexts where we observe sliding activity in adenovirus proteins. pVI, AVP–pVIc complexes and pVIc all bind to DNA with similar, low nM *K*_d(app.)_ values (Table 1); without pVIc, the *K*_d(app.)_ values for protein VI and AVP binding to DNA are ∼10-fold higher. AVP–pVIc complexes slide along DNA, but AVP alone, although it binds to DNA, does not slide. All the adenovirus sliding proteins, including pVIc, exhibit a similar binding footprint of ∼7 bp (Table 1 and Fig. 1b,c). Yet, these almost identical size footprints are from proteins whose molecular weights differ by more than a factor of 10. The pVIc moiety in the AVP–pVIc complex has been shown to interact with DNA; pVIc amino-acid residues are protected against oxidation in the presence, but not the absence, of DNA in synchrotron footprinting experiments^21^,^22^.

### Model for pVIc and AVP–pVIc complexes binding to DNA

**Figure 3:**
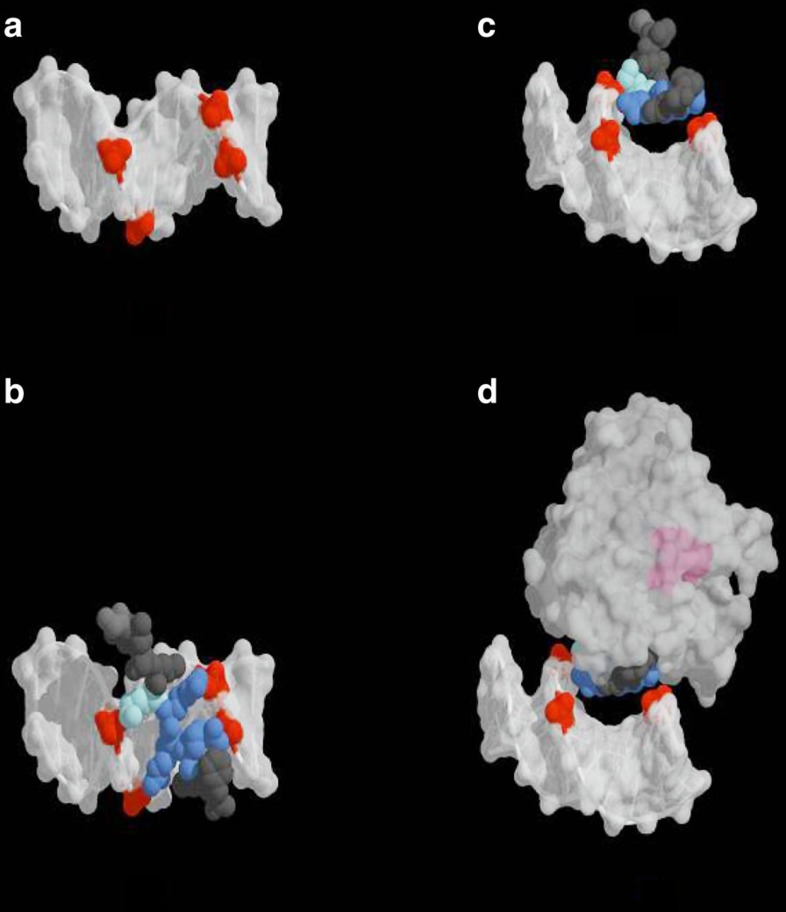
Model for the binding of pVIc and AVP–pVIc complexes to DNA. (**a**) The structure of a B-form DNA dodecamer (PDB ID: 1HQ7) is displayed with four of its phosphate groups coloured in red. (**b**) The structure of pVIc obtained from the crystal structure of the AVP–pVIc complex (PDB ID: 1NLN) is shown docked to the DNA. The four basic residues of pVIc, the one lysine residue and the three arginine residues, are coloured in light blue and dark blue, respectively. (**c**) The DNA-pVIc complex was rotated ∼90° on its *x* axis to show the contacts of the peptide with the major groove. (**d**) The AVP–pVIc structure is displayed, showing that DNA binding is dominated by the pVIc moiety. The majority of the binding enthalpy between the protein–peptide complex and DNA is likely to originate through electrostatic interactions between the four, contiguous, basic residues of pVIc (KRRR, in blue) in the AVP–pVIc complex and phosphate groups (in red) in the backbone of the dsDNA. The active site of AVP is coloured pink, showing that the proteinase active site is sterically unhindered by DNA binding, consistent with the proteinase being able to bind to and cleave substrates while bound to and sliding on DNA. The figure was rendered using PyMol (The PyMOL Molecular Graphics System, Version 1.2r.3pre, Schrödinger, LLC).

A model of pVIc bound to DNA that reflects the simplest interpretation of a body of disparate data, especially the ionic strength dependence of the binding of pVI^12^ and of AVP–pVIc complexes to DNA^14^, was generated by moving the crystal structure of the pVIc moiety in the AVP–pVIc complex^2^ on to the crystal structure of DNA, Fig. 3a. The only direct contacts are made by the four basic residues of pVIc, which form ion pairs^23^ with as many as four DNA phosphate groups, Fig. 3b,c. In the crystal structure of the AVP–pVIc complex, the four basic residues of pVIc are part of a β-strand; they are exposed to solvent^2^. Although we have placed pVIc in the major groove, we cannot rule out binding in the minor groove. If instead of placing the structure of pVIc taken from the atomic coordinates of the AVP–pVIc structure on to the structure of DNA, the structure of the entire AVP–pVIc complex is manually moved to the DNA, Fig. 3d, no additional contacts between the protein and DNA are made. Furthermore, the active site of AVP, coloured pink, shows that it is accessible to being able to bind substrate while the AVP–pVIc complex is bound to DNA. In this model, AVP–pVIc complexes bind to DNA solely via the pVIc moiety, more specifically via the four basic amino acids. These four amino acids are conserved (Fig. 1d).

### pVIc is a molecular sled

**Figure 4:**
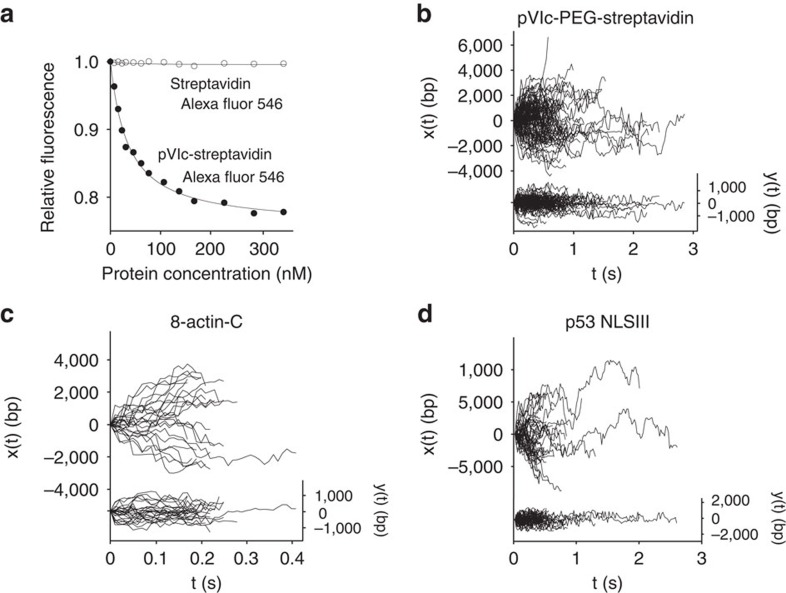
Sliding of heterologous proteins and peptides. (**a**) The equilibrium dissociation constant for the binding of (pVIc-biotin)-streptavidin to 18-mer dsDNA was determined by fluorescence resonance energy transfer. The quenching of the fluorescence intensity of the donor molecule, fluorescein-labelled 18-bp dsDNA, as a function of the concentration of the acceptor molecule (pVIc-biotin)-streptavidin Alexa Fluor 546 is shown by the closed circles. The relative fluorescence intensity is the fluorescence intensity at a specific concentration of acceptor divided by the initial fluorescence intensity of the donor in the absence of acceptor. The line through the closed circles represents the nonlinear regression fit of the experimental data to a 1:1 ligand–receptor model. The open circles represent data from the titration of 10 nM fluorescein-labelled 18-mer dsDNA with streptavidin Alexa Fluor 546. These data indicated that streptavidin Alex Fluor 546 did not bind to DNA. (**b**) (pVIc-biotin)-streptavidin complexes diffuse rapidly along DNA (*x*(t), left axis, 106 trajectories). Motion transverse to the DNA (*y*(t), right axis, is represented on the same scale, as a control. (**c**) A peptide (SIVHRKCF) with the last 8 amino acids of β-actin diffuses rapidly (69 trajectories) along DNA *x*(t). (**d**) The 13-amino acid peptide of NLSIII of the p53 protein (STSRHKKLMFKTE) diffuses rapidly along DNA at pH 6.5 (45 trajectories).

This model and extensive biochemical data suggest that pVIc may be a universal ‘molecular sled,' a vehicle capable of carrying any cargo attached to it along DNA. To test whether pVIc has general translocase activity, we attached a large, heterologous cargo, the streptavidin tetramer, to pVIc and assayed the complex for DNA binding and sliding activity. In a control experiment, streptavidin did not bind to DNA (Fig. 4a). However, pVIc–streptavidin complexes not only bound to DNA (Fig. 4a and Table 1), they diffused efficiently along DNA with a one-dimensional diffusion constant of 2.21±0.21 × 10^6^ (bp)^2^ s^−1^ (Fig. 4b and Table 1); the lower diffusion constant is consistent with the larger size of streptavidin tetramers compared with AVP, in their corresponding complexes with pVIc. Thus, pVIc is a ‘molecular sled' that can transport heterologous cargos along DNA. This is also the most convincing evidence that pVIc contributes the entirety of the sliding contacts between protein and DNA in all the contexts where we observe sliding activity in adenovirus proteins.

### Conservation and ubiquity of the pVIc amino-acid sequence

The four, contiguous basic amino acids of pVIc (KRRR) function not only in binding to and sliding on DNA^24^ but also function as a nuclear location signal (NLS)^25^. pVI binds to hexon, the major capsid protein of adenovirus and escorts it into the nucleus via the NLS in its pVIc moiety. The amino-acid sequence of pVIc is conserved among adenoviruses, Fig. 1d. Across human adenovirus serotypes, as well as among simian, porcine and murine adenoviruses, there is strict conservation of the KRRR motif within pVIc. Are there cellular proteins with sequences homologous to pVIc that slide on DNA? Since the NLS sequences of many nuclear-targeted proteins contain contiguous stretches of basic amino acids like those in the NLS in pVIc, is it possible that proteins that enter the nucleus of a cell via an NLS bind to and slide on DNA to interact with other proteins?

### C-terminal peptides from β-actin slide on DNA

Are there cellular proteins with sequences homologous to pVIc that slide on DNA? The C terminus of the major human protein β-actin, like pVIc, can stimulate AVP activity^26^,^27^. Of the last eight amino acids of β-actin (SIVHRKCF), four are identical and three are homologous to the last eight amino acids of pVIc (SLKRRRCF), Fig. 1d. The three contiguous basic residues at the C terminus of β-actin constitute the single longest stretch of basic residues in this acidic protein. Peptides comprised of the last 8 amino acids of β-actin, 8-actin-C and the last 11 amino acids of β-actin, 11-actin-C (SGPSIVHRKCF), bound tightly to DNA (Table 1) and diffused rapidly along it (Fig. 4c and Supplementary Fig. 3a,c). The MSD for each molecule was approximately linear (Supplementary Fig. 3b,d) yielding mean one-dimensional diffusion constants of 5.4–6.4 × 10^6^ (bp)^2^ s^−1^, Table 1. β-actin has been found in virus particles^28^ and in the nucleus of cells^29^ and has been shown to be involved in chromatin remodelling^30^ and gene activation^31^.

### Peptide with an NLS from the p53 protein slides on DNA

Are there proteins that bind to and slide along DNA using an NLS as a molecular sled? The extremely important tumour-suppressor protein p53 has been shown to slide on DNA^32^,^33^,^34^, and sliding activity has been localized to its C terminus. p53 contains three functional NLSs, including two in the C-terminal domain (CTD; aa 363–393). A 13-amino acid peptide fragment of the p53 CTD (STSRHKKLMFKTE, aa 376–388) containing NLSIII (RHKKLM) binds to DNA (Table 1) and exhibits robust sliding activity with a measured mean diffusion constant of 11.2±0.8 × 10^6^ (bp)^2^ s^−1^ (Fig. 4d, Table 1 and Supplementary Fig. 4a,b). Thus a peptide with an NLS from a cellular protein can bind to and slide along DNA.

## Discussion

Here we asked the question: by what mechanism do two adenovirus proteins, a substrate and an enzyme, slide along DNA to form productive collisions, collisions that lead to binding? We showed they slide via pVIc, an 11-amino acid peptide. pVIc itself binds to DNA with a nM equilibrium dissociation constant; binding is independent of DNA sequence; once bound, pVIc covers 6–7 base pairs^14^ of DNA, Table 1. Surprisingly, pVIc slides by itself on DNA exhibiting the fastest one-dimensional diffusion constant yet observed, 26±1.8 × 10^6^ (bp)^2^ s^−1^ with an s.d. of 11 × 10^6^ (bp)^2^ s^−1^ (ref. 14). Is pVIc a ‘molecular sled' capable of carrying cargos such as AVP or protein VI and possibly heterologous proteins or does it induce sliding by the adenovirus proteins by inducing a conformational change that enables them to slide? Streptavidin does not bind to DNA, yet a streptavidin-pVIc complex not only binds to DNA but also slides along DNA. Thus pVIc is a ‘molecular sled' that can slide not only adenovirus proteins along DNA but also heterologous cargos along DNA. A peptide similar in sequence to pVIc, the C terminus of β-actin, was shown to slide on DNA. A model is presented that depicts pVIc binding to DNA via ion pairs formed between its four basic amino-acid residues and four phosphate groups on DNA. These four basic amino acids of pVIc constitute a functional NLS^25^ raising the possibility that proteins with similar NLSs slide on DNA. We showed that a peptide containing NLSIII from the p53 protein slides on DNA.

That pVIc slides on DNA by itself was a surprise; the 11-amino acid peptide lacks the large, structured DNA-binding interface present in proteins previously shown to slide on DNA^1^,^2^,^28^,^31^,^35^. Consistent with this is the observation that in the crystal structure of the AVP–pVIc complex, pVIc is tightly bound to AVP^1^,^2^. There are 24 non-β-strand hydrogen bonds, 6 β-strand hydrogen bonds and one-covalent bond. A disulfide bond is formed between Cys104 of AVP and Cys10′ of pVIc. Structured DNA-binding interfaces have been presumed necessary to achieve the low free-energy barriers to sliding (comparable to k_B_T) that are needed for effective searching along DNA^15^,^36^. This is understood to be achieved in sequence-non-specific protein–DNA complexes by indirect or frustrated protein–DNA contacts that ‘lubricate' the binding interface. Such ‘buffered' protein–DNA contacts are thought to support rapid sliding by minimizing differences in free energy as the protein translates along the DNA and have been observed in protein–DNA complexes (interfacial water^37^, long-range interactions, for example, between helix dipoles and the DNA^38^, topological linkage to the DNA^39^, and frustrated ‘interleaved'^40^ or ‘out of phase'^41^ binding interactions, or a large number of weak binding interactions^40^). One-dimensional diffusion constants for some proteins are known to be sensitive to point mutations and the presence of individual charged residues^16^,^42^. Together, these observations established the expectation that precision-tuned, three-dimensional structures are required for high-speed sliding. However, we observed both robust and persistent sliding on DNA by pVIc, a peptide predicted by several secondary structure prediction programs to have no stable secondary structure. The mean one-dimensional diffusion constant of pVIc was the highest yet reported for any object sliding along DNA, 26±1.8 × 10^6^ (bp)^2^ s^−1^ (Table 1)^14^.

The differences in the slopes in MSD plots from different pVIc molecules were much greater than the uncertainty in the slope of a plot for an individual molecule; this indicates that the distribution arose from dispersion in the diffusion constants for different, individual pVIc–DNA complexes and not entirely from measurement error. For AVP–pVIc and (pVIc–biotin)-streptavidin, the mean one-dimensional diffusion constants were 21.0 × 10^6^ with s.d. of 15.6 × 10^6^ (bp)^2^ s^−1^ and 2.21 × 10^6^ with s.d. of 1.99 × 10^6^ (bp)^2^ s^−1^, respectively. The distribution of one-dimensional diffusion constants for pVIc was more narrow, with mean 26.0 × 10^6^ and s.d. of 11 × 10^6^ (bp)^2^ s^−1^ (Fig. 2d and Table 1). Perhaps the wide distribution of one-dimensional diffusion constants reflects the presence of many, slightly different polypeptide conformations that persist for seconds upon binding to the DNA; this would be consistent with the initial sliding rates being different but constant. Among a growing literature of examples of conformational heterogeneity^43^,^44^,^45^, it was recently shown that there are >40 slightly different conformations of ubiquitin in dynamic equilibrium and that binding of other proteins to ubiquitin occurs via conformational selection^46^.

The one-dimensional diffusion constant for the pVIc–streptavidin complex, Table 1, is consistent with our physical model for the dependence of this constant on the protein's size (radius R) and the offset of its centre of mass from the DNA axis (offset R_oc_)^15^,^18^. The model was used to analyse the diffusion constants of AVP–pVIc, hOgg1 and hOgg1–streptavidin^14^,^15^,^16^. The one-dimensional diffusion constants of these proteins all scaled as 1/(R^3^+3/4R(R_oc_)^2^). hOgg1–streptavidin and pVIc–streptavidin were each conjugated using a PEG 3K linker, which by itself has a predicted size comparable to streptavidin. The attachment point of the PEG linker to the hOgg1 C terminus is close to the DNA, making the magnitudes of the R and R_oc_ parameters of the hOgg1–streptavidin complex very similar to those of the pVIc–streptavidin complex. The diffusion constant of the hOgg1–streptavidin conjugate was measured as 2.2 × 10^6^ (bp)^2^ s^−1^. This value is consistent with the prediction from the physical model as were the one-dimensional diffusion constants of the pVIc–streptavidin complex and the AVP–pVIc complex (Table 1)^14^.

How do AVP–pVIc complexes and pVIc bind to DNA? Experiments on the ionic strength dependence of the sliding of AVP–pVIc complexes and of pVI on DNA are consistent with the pVIc moiety mediating the binding to DNA. The model in Fig. 3 depicts the sliding interface as consisting of the four contiguous basic amino acids of pVIc forming ion pairs with up to four phosphate groups on the DNA. The model is consistent with a binding footprint on DNA of ∼7 bp for pVIc, the AVP–pVIc complex and pVI (Table 1). The major factor driving the non-specific interaction between AVP–pVIc complexes^10^ or pVI^12^ and DNA comes from the entropic contribution on the release of counter ions. The sequence-non-specific interactions between AVP–pVIc complexes or pVI and DNA exhibit a substantial dependence on the monovalent sodium ion concentration. This dependence reflects the electrostatic component of the binding reaction^47^. The electrostatic component originates from the formation of ion pairs between positively charged groups on the AVP–pVIc complex or on pVI and negatively charged phosphate groups on DNA. After binding occurs, there is a concomitant release of counter ions from the DNA and, possibly, from AVP–pVIc complexes or pVI. From an analysis of the equilibrium association constants for the binding of AVP–pVIc complexes or pVI to 12-mer dsDNA as a function of the Na^+^ concentration, an accurate estimate of the number of ion pairs involved in the interaction was obtained. For AVP–pVIc complexes and for pVI, 2.2 (ref. 10) and 2.9 (ref. 12) ion pairs, respectively, were involved in complex formation with 12-mer dsDNA.

How does pVIc slide on DNA? Dynamic formation and breakage of the ionic contacts may explain the observed motility and processivity of AVP–pVIc complexes and pVIc on DNA^48^. During movement of the sled along DNA, one or more of the four potential ion pairs could break and re-form elsewhere; concomitantly, the other ion pairs could remain intact, holding the moving peptide to at least one part of the DNA at all times. This is consistent with the average of 2–3 ion pairs observed in pVIc-mediated protein–DNA complexes^10^,^12^. Multiple binding sites used simultaneously could explain the high processivity observed in these single-molecule translocation experiments. We had also previously shown that sliding of AVP–pVIc complexes occurs along a helical path (Supplementary Note 4) with the protein complex rotating as it moves so that its DNA-binding face maintains contact with the DNA^15^.

The model for the binding of pVIc and AVP–pVIc complexes to DNA explains a long-standing enigma about the crystal structure of the AVP–pVIc complex: Why is pVIc which exerts powerful control on the rate of catalysis, bound quite far from the active-site residues involved in catalysis? A secondary structure model of the AVP–pVIc complex with the atoms of the pVIc residue colour coded is shown in Supplementary Fig. 1. The pVIc cysteine residue, which forms a disulfide bond with Cys104 of the main chain, is 32 Å away from the active-site nucleophile Cys122. The residue of pVIc closest to the active site is Val2′, whose side chain is 14.5 Å from Cys122. Heretofore, it had been thought that the only function of pVIc was to act as a cofactor by increasing the catalytic power of the active site. However, knowing a second function of pVIc, as described here, namely sliding of AVP along DNA, it became clear why the pVIc-binding site must be far away from the active site: if the active site of AVP were close to pVIc bound to DNA, that configuration may physically interfere with the ability of the active site to recognize and cleave a precursor protein also bound to the same DNA molecule. Separation of the active site from pVIc enables the enyzme active site to interact readily with its seven, different substrates.

Current views on one-dimensional diffusion of proteins along DNA are mostly concerned with proteins that locate and bind to a specific site on DNA, proteins involved in DNA metabolism. Ionic strength is thought to be a major factor in determining the search dynamics. For example, in low salt *in vitro*, the lac repressor binds to its operator with a second-order rate constant of 7 × 10^9^ M^−1^ s^−1^ (ref. 49). However, in the presence of 100 mM KCl, the rate falls 100-fold^50^, to the diffusion-limited rate^51^,^52^. The reason given for this extremely fast interaction at low ionic strength is that there is an electrostatic attraction between a positively charged site on the repressor and the negatively charged phosphate groups on the DNA. The decrease in the association rate at physiological ionic strength is due to the solvent counter ions screening the electrostatic attraction. From this, it was concluded that at physiological ionic strength the rate-determining step in binding to a specific DNA site is the diffusion of the protein to the DNA molecule, not the transfer of the protein molecule from a non-specific site to a specific site^53^.

Here we are concerned with how and why some adenovirus proteins inside the virion at physiological ionic strength locate and productively bind to other proteins via one-dimensional diffusion on the viral DNA. What is the rate-determining step in this environment? Our sliding assays (Supplementary Discussion) were done under non-physiological conditions, at low ionic strength. Raising the ionic strength to physiological conditions (0.15 M NaCl) will decrease the association rate of a protein binding to DNA; however, the enormously high concentration of DNA in the virion^54^ will increase the association rate of a protein binding to DNA. The second-order rate constant for binding of a protein to DNA is proportional to the DNA binding site concentration, which is huge, 0.5 M in the virion. Also, increasing the ionic strength to physiological conditions will have a minimal effect on the one-dimensional diffusion constant for sliding along DNA as that process is insensitive to ionic strength.

So, what are the factors that determine the three-dimensional rate of diffusion of pVI and AVP–pVIc complexes in the virion? Certainty the rate of diffusion in three-dimensional space is much faster, by orders of magnitude^18^,^53^, than it is in the one-dimensional space on the DNA contour. But, is that effectively true for some adenovirus proteins inside the adenovirus virion? It is not. Inside the virion, two factors act together to reduce the effective three-dimensional bimolecular association rate between the proteinase and its substrates. The high concentration of DNA drives these sequence-independent DNA-binding proteins (the proteinase and its seven different substrates)^4^,^55^ onto the DNA by mass action. For example, in an adenovirus virion, the concentration of DNA-binding sites for AVP–pVIc complexes is ∼500 mM (ref. 54), and the *K*_d_ for the binding of AVP–pVIc complexes to DNA at 0.15 M NaCl (ref. 10) is 10 μM. Therefore at equilibrium, the ratio of protein free to protein bound in the virus particle, (*K*_d_)(DNA)^−1^, is 2 × 10^−5^. This means that at any one time, for each AVP–pVIc molecule not bound to DNA, there will be 50,000 AVP–pVIc molecules bound to and presumably sliding along DNA. In a virion with 50 molecules of AVP^13^ only 0.001 molecules of AVP will be free; 99.998% of the 50 AVP–pVIc complexes will be bound. Thus, most of the time, almost none of the AVP–pVIc complexes is free in solution in a given virion. And this would diminish, by lowering the duty cycle for three-dimensional diffusion, the ‘effective' three-dimensional diffusion constant by a similar factor, 50,000-fold. A similar situation occurs with pVI. At an NaCl concentration of 0.001 M, the estimated *K*_d_ for the binding of pVI to DNA is 0.027 nM (ref. 12); this increases >350,000-fold, to 9.5 μM, at physiological ionic strength. However, at 0.15 M NaCl, the ratio of protein free to protein bound, (*K*_d_)(DNA)^−1^ in the virus particle, is 1.9 × 10^−5^.

Even though at high ionic strength the residence time per event will be decreased, the net amount of time a protein is sliding on DNA will be quite large. This is because there will be numerous short sliding events, over hundreds of base pairs, as opposed to fewer but longer sliding events. An added benefit of high ionic strength and DNA concentration is the amount of unique DNA scanned per unit time by sliding; this will be increased since, presumably, hopping or jumping will occur after each dissociation event thereby reducing the redundancy of the one-dimensional search. This balance between one-dimensional searching and dissociation/rebinding would have the additional benefit of enabling efficient bypass of obstacles on the DNA.

The second factor that reduces the effective three-dimensional bimolecular association rate between the proteinase and its substrates is the mesh size of the DNA matrix. We calculate the mesh size of the DNA matrix inside the virion to be <1 nm (ref. 48). Three-dimensional diffusion was shown to be reduced by as much as an order of magnitude when the mesh size of a dynamic polymer network equals the size of the diffusing object. The AVP–pVIc complex is ovoid with dimensions of 41 × 44 × 55 Å (refs 1, 2). Since the diameter of the AVP–pVIc complex is much larger than the mesh size, and the DNA network has restricted mobility by being packed in the small volume of the virion, a further factor of 10 in the reduction of the diffusion constant is a conservative estimate as to the effect of the DNA matrix on the three-dimensional diffusion of AVP–pVIc complexes.

And, there is no other way of compensating for these reductions in the effective diffusion rate. For example, the DNA genome cannot move to promote protein–protein interactions as it is jammed inside the virion because of being packaged under high pressure. The pressure exerted by the DNA on the virion walls, possibly in excess of 100 atm (ref. 56), results in a large friction between the DNA and the inner surface of the virion, freezing the DNA in place and rendering DNA-bound proteins likewise immobile.

Together, the enormous DNA concentration in the virion, which diminishes the three-dimensional diffusion constant by 50,000-fold and the mesh size of the DNA, which diminishes the three-dimensional diffusion constant by a factor of 10, indicate the ‘effective' three-dimensional diffusion constant would be reduced by a factor of over 500,000. Thus, the reason these adenovirus proteins form productive collisions via one-dimensional diffusion along DNA is that they have no choice; doing so by three-dimensional diffusion is impossible. In this case, the rate-determining step is not the diffusion of a protein molecule to a DNA molecule but the transfer of a molecule along the DNA to its target protein.

A similar situation probably exists in the nucleus of mammalian cells and in the nucleoid of bacterial cells, where the concentration of DNA binding sites is also high. A discussion of initial diffusion-limited reaction rates among protein molecules and approximate lower limits of time to react half the molecules in a population of molecules under different DNA-binding and one-dimensional diffusion scenarios is presented in Supplementary Table 1 and in Supplementary Discussion.

The data shown here that elucidated the mechanism of sliding of two adenovirus proteins have several interesting and potentially important implications. The discovery that short, basic peptides can confer a DNA binding and sliding activity on a protein vastly increases the possible number and types of proteins that may bind to DNA nonspecifically and exhibit sliding activity. Tight DNA binding is not necessary for effective sliding activity. Since the concentration of DNA binding sites in the nucleus of mammalian cells and the nucleoid of bacterial cells is high (similar to that in adenovirus virions), even proteins with short basic sequences that have weak, non-specific DNA-binding affinity will be bound most of the time to DNA, being driven there by the DNA concentration. For example, that pVIc with its NLS sequence (KRRR) binds to DNA and slides AVP–pVIc complexes and pVI on DNA and that NLSIII from the p53 protein binds to and slides along DNA imply that possibly all proteins that enter the nucleus of a cell with similar, basic NLSs may bind to and slide along DNA. If so, this will have a significant effect on protein–protein association rates in these environments. Presumably all NLSs must be on the surface of a protein to be able to be recognized by the nuclear import apparatus. That the activity of ‘molecular sleds' seems to be encoded purely in their primary sequences raises the tantalizing possibility that DNA binding and sliding activity may be predicted from genomic data. This is how we discovered the sliding activity at the C terminus of β-actin and NLSIII of the p53 protein.

To date, one-dimensional diffusion along DNA has been found almost exclusively within the purview of proteins that search for a specific locus on the DNA genome. We had previously observed that the one-dimensional compartment along DNA can also support, promote and regulate transactions between proteins. For example, sliding on DNA is required for the activation of AVP by pVI. If only a simple bimolecular interaction between AVP and pVI were needed for activation, then mixing purified AVP^7^ with purified pVI^12^ should result in the cleavage of pVI to yield pVIc followed by the formation of active AVP–pVIc complexes. However, when this was done, no enzymatic activity was detected, even at μM concentrations^11^ of AVP and pVI. DNA is required for the activation of AVP by pVI. Both AVP and pVI bind to DNA with apparent equilibrium dissociation constants of 63 nM (ref. 10) and 46 nM (ref. 12), respectively. When we repeated the experiment but in the presence of dsDNA^11^, by 1 h, 100% of the pVI was cleaved and used to form active AVP–pVIc complexes. Thus, activation of AVP to AVP–pVIc complexes by pVI required the presence of DNA. Furthermore, AVP and pVI both must be on the same DNA molecule for activation to occur as activation can only occur in *cis*, not *trans*^11^. Thus, sliding along DNA is required for the activation of AVP by pVI to AVP–pVIc complexes.

That protein–protein interactions can be regulated by DNA binding and one-dimensional diffusion greatly increases the scope of biochemistry mediated by facilitated diffusion on DNA: it also has implications for biotechnology^57^,^58^. Targeting protein–protein interactions to the DNA genome localizes their interaction in the cell. And DNA might be more than just a ‘highway' accelerating reactions. It could have a dramatic effect on the specificity of protein–protein interactions by introducing new mechanisms^59^,^60^. On the DNA contour, bimolecular interactions may not only be regulated by one-dimensional diffusion constants, but also by the relative orientations of the DNA-bound proteins. If, for example, an enzyme and its substrate become optimally orientated for a productive collision when both are bound to the same DNA helix, the fraction of productive enzyme-substrate collisions mediated by sliding may be orders of magnitude greater, possibly as high as 1/1, far higher than in the case of the random orientation collisions that occur in three-dimensional space^31^.

In conclusion, we have identified and characterized the physical mechanism by which two adenovirus proteins slide on DNA. They use a ‘molecular sled,' a new vehicle in molecular biology, an 11-amino acid peptide that slides on DNA by itself or, when attached to a heterologous cargo, can carry the cargo along DNA. Sliding along DNA is the only way sequence-independent DNA-binding proteins can interact with each other in certain milieus, for example, inside nascent virions they are essentially irreversibly bound to the viral DNA, driven there by the enormous DNA concentration. The biological relevance of our data is that, for example, proteins in the nucleus of cells that contain even just short clusters of basic amino acids would be predicted to be essentially irreversibly bound to the nuclear DNA and would be able to interact with other nuclear proteins only by sliding along the DNA. Characteristics of the ‘molecular sled' in its milieu, for example, virion or nucleus, have implications for how proteins can interact and imply that a new form of biochemistry, one-dimensional biochemistry, is operative.

## Methods

### Materials

pVIc (GVQSLKRRRCF), streptavidin Alexa Fluor 546 conjugate, 5′-fluorescein-labelled 12-mer ssDNA (5′-GACGACTAGGAT-3′), 5′-fluorescein-labelled 18-mer ssDNA (5′-CAGGAAACAGCTATGACC-3′), and 5′-fluorescein-labelled-36-mer ssDNA (5′-GATTGCATGATTAGAGTGTGCTGGATGTGATAGTGA-3′) were purchased from Invitrogen (Carlsbad, CA). Labelled ssDNAs were annealed to their complimentary strands according to standard protocols. 8-Actin-C (SIVHRKCF) and 11-Actin-C (SGPSIVHRKCF) were purchased from Research Genetics (Huntsville, AL, USA). Cy3B-maleimide was purchased from GE Healthcare (Piscataway, NJ). Biotin PEG-3K maleimide was purchased from Nektar. Octylglucoside was purchased from Fisher Scientific (Faden, NJ). *n*-dodecyl-β-D-maltopyranoside (DDM) was purchased from Anatrace (Maumee, OH). The concentrations of pVIc, 8-Actin-C and 11-Actin-C were determined by titration of the cysteine residue with Ellman's reagent^61^,^62^ using an extinction coefficient of 14,150 M^−1^ cm^−1^ at 412 nm for released thionitrobenzoate. The N-terminal TMR-labelled human p53 CTD segment (AA 376–388) was purchased from the Biopolymer & Proteomics Laboratory at MIT (>85% purity).

### Binding of pVIc to DNA

The equilibrium dissociation constant, *K*_d(app.)_ for the binding of pVIc to DNA was determined by incubating 10 nM Cy3B-pVIc in 20 mM HEPES (pH 7), 10 mM NaCl, 0.025% DDM, and 0.1 mM dithiothreitol (DTT) with increasing amounts of 12-mer dsDNA and measuring the change in anisotropy. The line through the closed circles in Fig. 1a represents the nonlinear regression fit of the experimental data to a 1:1 ligand–receptor model. The *K*_d(app.)_ was 264±25 nM (ref. 14). The stoichiometry of binding of pVIc to DNA was determined by incubating 10 nM Cy3B-pVIc and 10 μM pVIc with increasing amounts of 36-mer dsDNA in 10 mM Tris-HCl (pH 8) and 2 mM β-octylglucoside and measuring the change in anisotropy, Fig. 1b. The two straight lines were drawn using the data in the filled-in circles. The point with the open circle was not included in the fits to the lines. The intersection point of the two lines is the minimal concentration of DNA required to saturate 10 μM pVIc; it indicates six molecules of pVIc saturate one molecule of 36-mer dsDNA. The number of base pairs of DNA occluded by the binding of one pVIc to DNA: the experiment in Fig. 1b was repeated but with 12-mer and with 18-mer dsDNA, and the stoichiometries of binding plotted versus the DNA length in base pairs, Fig. 1c. The line indicates that one molecule of pVIc occupies 6.6 bp of dsDNA. For determining the *K*_d_ of the 13-amino acid peptide containing NLSIII from the p53 protein, the fluorophore-labelled peptide concentrations were kept constant at 10 nM, and the 30 bp dsDNA concentrations ranged from 0 to 3 μM.

### Sliding assay

The sliding assay, based on total internal reflection fluorescence microscopy, used phage λ DNA molecules (48,502 bp) attached at one end to a glass cover slip surface by a biotin linkage^35^. A flow cell was constructed on top of the cover slip such that when a laminar flow of buffer is applied, the DNA stretches in the direction of flow and orients itself parallel with and in close proximity to the surface of the cover slip. Evanescent waves (532 nm) from a LASER reflecting off the coverslip-buffer interface are used to illuminate a very small volume (∼1 pl) within a few hundred nanometres of the glass surface. The interaction of a single, fluorescently labelled protein with DNA was visualized with low fluorescence background by wide-field microscopy using a sensitive CCD camera (Photometrics Cascade 512:B) or scientific CMOS (Hammamatsu Orca Flash 4.0). Despite the optical resolution limit imposed by diffraction, the centroid position^14^ of each signal in each frame is determined with sub-diffraction-limited resolution, typically 10–30 nm for bright fluorescent conjugates.

Labelled peptides or proteins were infused at concentration of 1–2 nM at rates of 20–50 ml h^−1^. High flow rates were chosen to drive the longitudinal DNA fluctuation faster than the imaging frame rate^16^. The assay buffer consisted of 10 mM MES (pH 6.5), 2–25 mM NaCl, 50 μM EDTA, 20 mM ethanol, 5% (v/v) glycerol and 5 mM DTT or β-mercaptoethanol. ‘Low salt' measurements were conducted with 2–6 mM NaCl; ‘higher salt' measurements were conducted with 20–25 mM NaCl.

### Binding of (pVIc)·streptavidin complexes to DNA and sliding

In Fig. 4a, the equilibrium dissociation constant for the binding of (pVIc-biotin)-streptavidin to 18-mer dsDNA was determined by fluorescence resonance energy transfer. The quenching of the fluorescence intensity of the donor molecule, fluorescein-labelled 18-bp dsDNA, as a function of the concentration of the acceptor molecule (pVIc-biotin)-streptavidin Alexa Fluor 546 is shown by the closed circles. The relative fluorescence intensity is the fluorescence intensity at a specific concentration of acceptor divided by the initial fluorescence intensity of the donor in the absence of acceptor. The line through the closed circles represents the nonlinear regression fit of the experimental data to a 1:1 ligand–receptor model. The open circles represent data from the titration of 10 nM fluorescein-labelled 18-mer dsDNA with streptavidin Alexa Fluor 546. The data indicated that streptavidin Alex Fluor 546 did not bind to DNA. Both curves were corrected for inner filter effects, which were <10% at the highest concentration of acceptor. In Fig. 4b, (pVIc-biotin)-streptavidin complexes diffuse rapidly along DNA (*x*(t), left axis, 106 trajectories). Motion transverse to the DNA (*y*(t), right axes) is represented on the same scale, as a control.

### Fluorescent labelling of peptides

The cysteine side chains of pVIc, 8-Actin-C, 11-Actin-C were labelled at a concentration of 200 μM in 25 mM HEPES (pH 7.0), 25 mM NaCl, and 20 mM ethanol by the addition of Cy3B-maleimide to 600 μM. Labelling reactions were incubated at 21 °C in the dark for 2.5 h. Dye-conjugated peptides were purified from unreacted dye and peptide on a 15 cm × 4.6 mm Discovery C18, 5 μm column. Peptides were eluted via a linear acetonitrile gradient from 0 to 40% in 0.1% trifluoroacetic acid. The conjugated peptide peak was identified by MALDI-TOF analysis. The fractions were evaporated to dryness on a LABCONCO Centrivap Console and resuspended in water. The concentration of the dye-conjugated peptide was determined by measuring the dye concentration spectrophotometrically using 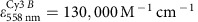. The conjugated peptide was aliquoted, dried and stored at –20 °C.

### Alexa fluor 546-streptavidin-biotinylated pVIc conjugates

Alexa Fluor 546-streptavidin-biotinylated pVIc conjugates were formed as follows: pVIc was biotinylated by incubating 3.4 mM pVIc in 100 mM sodium phosphate (pH 7.0) and 20 mM ethanol with 7.8 mM biotin-PEG-3K maleimide in the dark for 16 h at room temperature. The reaction was quenched by the addition of DTT to 10 mM. The biotinylated pVIc was purified on a Discovery C18 column equilibrated in 0.1% (v/v) trifluoroacetic acid using a linear gradient of acetonitrile. Fractions off the column that stimulated AVP activity and were unreactive to Ellman's reagent were pooled. Next, ∼850 pmol of the biotin-PEG-pVIc were serially diluted 1:2 with acetonitrile and each dilution lyophilized to dryness using a Labconco Centrivap. Then, a constant volume of 16.7 μM Streptavidin Alexa Fluor 546 was added resulting in molar ratios of (pVIc-biotin) to streptavidin Alexa Fluor 546 of 1:1, 2:1, 4:1, 8:1 and 16:1. After incubation for 1 h, the conjugates were stored at 4 °C.

### Fluorescence anisotropy

Steady-state fluorescence anisotropy measurements were performed using an ISS model PC-1 photon counting spectrofluorometer (ISS, Champaign, IL) equipped with polarization accessories or a SpectraMax M5 plate reader from Molecular Devices. Measurements on the PC-1 instrument were made in L-format using a 300-W xenon arc lamp with 10 and 14 mm Glan-Thompson polarizers in the excitation and emission channels, respectively. For the Cy3B dye, the excitation wavelength was 564 nm, with 8 nm slits placed before and after a monochromator. The parallel and vertical emission components were measured through a 580-nm bandpass filter with a FWHM of 10 nm. For fluorescein dye, the excitation wavelength was 495 nm, with 8 nm slits placed before and after a monochromator. The parallel and vertical emission components were measured through a 530-nm longpass filter. For measurements on the M5 instrument, samples were prepared in a 96-well plate, and the excitation/emission/cutoff wavelengths were set at 544/590/570 nm (alternatively 590 nm cutoff; both cutoff settings generating substantially identical *K*_d_ values from fits).

### Calculation of *K*_d_ values

The *K*_d_ was calculated by fitting the fluorescence anisotropy data to a one-to-one stoichiometry binding model according to the equation:


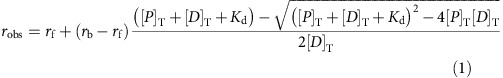


where *r*_obs_ is the observed anisotropy; *r*_f_ the anisotropy of free 12-mer ssDNA; *r*_b_ the anisotropy of protein bound DNA; [*P*]_T_ the total protein concentration; [*D*]_T_ the total DNA concentration (10 nM); and *K*_d_ is the equilibrium dissociation constant. The parameters in the nonlinear regression analysis were *r*_f_, *r*_b_ and *K*_d_.

### Fluorescence resonance energy transfer

Steady-state fluorescence measurements were measured with an ISS PC-1 spectrofluorometer (ISS, Champaign, IL) with a 300-W xenon arc lamp and 19 amp lamp current. The excitation and emission wavelengths were 490 and 520 nm, respectively, using 8 nm excitation and emission slits. A 1 ml solution of 10 nM fluorescein-labelled 18-mer dsDNA in 20 mM sodium phosphate, pH 7.5, 0.05% DDM was placed inside a 1 cm standard quartz cuvette and was titrated with increasing amounts of streptavidin Alexa Fluor 546 or the complex (pVIc-biotin).streptavidin Alexa Fluor 546. The solution was mixed and allowed to equilibrate for 2 min before measuring the fluorescence intensity for a maximum of 10 s (an average of 11 measurements). To correct for the decrease in fluorescence intensity due to inner filter effects, a 10-nM solution of fluorescein was titrated with streptavidin Alexa Fluor 546.

### Centroid determination and analysis of trajectories

Commercial particle-tracking software (Diatrack 3.0, Semasopht, Switzerland) and custom particle-tracking code developed in house were used to identify individual DNA-bound protein molecules and track their trajectories. Molecules exhibiting transverse displacements consistent with flow-stretched DNA were included in the analyses, while molecules exhibiting trajectories characteristic of surface adsorption were excluded. Molecular trajectories were analysed in Matlab by methods similar to those previously published^16^.

## Additional information

**How to cite this article:** Mangel, W. F. *et al.* Molecular sled is an eleven-amino acid vehicle facilitating biochemical interactions via sliding components along DNA. *Nat. Commun.* 7:10202 doi: 10.1038/ncomms10202 (2016).

## Supplementary Material

Supplementary Information Supplementary Figures 1-4, Supplementary Table 1, Supplementary Notes 1-4, Supplementary Discussion and Supplementary References

## Figures and Tables

**Table 1:** One-dimensional diffusion constants (*D*_1_), equilibrium dissociation constants (*K*_d_) and size of binding site on DNA.

**Protein (bp DNA)** ***M***_**W**_ **(aa)**	***D***_**1**_**±s.e. (bp)**^2^ **s**^−1^** × 10**^−6^	**s.d.** ***D***_**1**_ **(bp)**^2^** × 10**^−6^	***K***_**d(app.)**_**nM (pH)**	**DNA footprint (bp)**	**Notes**
pVIc (12) 1,350 (11)	ND	ND	693±84 (pH 8)	7	1, 2, 3
pVIc(low salt)	26.0±1.8	11	264±25 (pH 7)	7	1, 4, 5
pVIc(high salt)	17.9±3.5	10.7	ND	ND	1, 4
AVP (12) 23,087 (204)	(0.02±0.07)		63.1±5.8 (pH 7)	ND	1, 3, 6, 7
AVP–pVIc (36) 24,435 (215)	21.0±1.9	15.6	4.6±2.2 (pH 7.5)	6	1, 3, 5, 8
AVP–pVIc (high salt)	17.1±3.5	16.2	ND	ND	1, 4, 5
(pVIc-biotin: streptavidin) (18) ∼70,000 (651)	2.21±0.21	1.99	35±5.0 (pH 7.5)	ND	1, 8
pVI (33) 27,014 (250)	1.45±0.13	1.61	46±1.6 (pH 8)	8	1, 2, 7, 9
Protein VI (33) 22,118 (206)	ND	ND	307±38 (pH 8)	ND	1, 2, 9
8-Actin-C (12) 988 (8)	5.45	3.63	5.0±0.8 (pH 7)	ND	1
11-Actin-C (12) 1,230 (11)	6.40	3.29	ND	ND	1
13-p53-C (30) 1,593 (13)	11.2±0.8	3.5	780±96 (pH 7.4)	ND	1, 10

AVP, Adenovirus proteinase; ND, not determined; pVIc, peptide derived from C terminus of pVI, the precursor to protein VI; pVI, Precursor to adenovirus protein VI; Protein VI, Adenovirus protein VI.

Notes: (1) to convert from bp to nm: 10^6^ (bp)^2^ s^−1^=102,400 (nm)^2^ s^−1^. For *K*_d(app.)_ determinations, at pH 7.5 or 8, the dye was fluorescein, and the label was on the DNA; at pH 7 the dye was Cy3B and the label was on the protein. For pVIc-biotin: streptavidin experiments at pH 7.5, the dye used was Alexa Fluor 546, and there were two dye molecules per streptavidin. pVIc was labelled with Cy3B at Cys10′. AVP and AVP–pVIc were labelled with Cy3B at Cys199. The actin C-terminal peptides were labelled on their cysteine residue with Cy3B. pVI was labelled at Cys249 with Cy3B. The p53 CTD segment (aa 376–388) was labelled at its N terminus with tetramethyl rhodamine; (2) assay buffer was 20 mM Tris-HCl, pH 8, 0.025% DDM, 0.1 mM DTT; (3) ref. 10; (4) Binding assay buffer was 20 mM HEPES, pH 7, 0.025% DDM, 10 mM NaCl and 0.1 mM DTT. The NaCl concentration in the sliding assay buffer was 2–6 mM NaCl; in the high salt-sliding assay buffer, 20–25 mM NaCl was present (see Supplementary Text); (5) ref. 14; (6) whole population-mean *D*_1_ calculated from one population (99–96% of the molecules bound to DNA) having a *D*_1_ of zero and another population (1–4% of the molecules bound to DNA) having a *D*_1_ of 1.7 × 10^6^ (bp)^2^ s^−1^, with s.d. of 1.9 × 10^6^ (bp)^2^ s^−1^; (7) ref. 11; (8) assay buffer was 20 mM Na phosphate, pH 7.5, 0.05% DDM; (9) ref. 12, (10) Assay in 10 mM sodium phosphate buffered at pH 6.5 or 7.4.
