# Supplementary material for: Molecular sled is an eleven-amino acid vehicle facilitating biochemical interactions via sliding components along DNA
Source: Nat Commun. 2016 Feb 2;7:10202. doi: 10.1038/ncomms10202 (PMC4740752; doi:10.1038/ncomms10202)
Supplement: Supplementary Information — Supplementary Figures 1-4, Supplementary Table 1, Supplementary Notes 1-4, Supplementary Discussion and Supplementary References [file ncomms10202-s1.pdf]

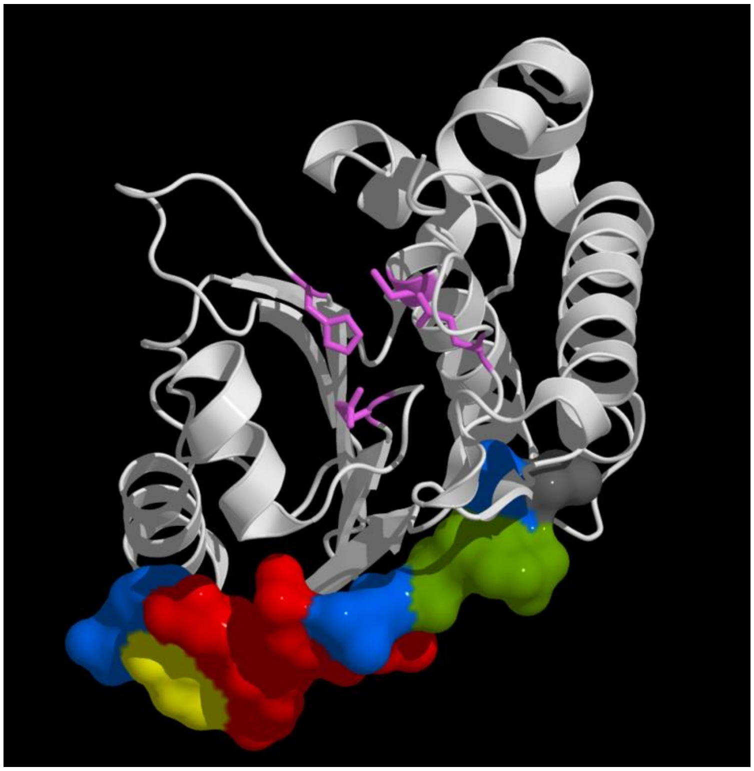

Supplementary Figure 1 l Crystal structure of the AVP-pVIc complex. In the crystal structure of the AVP-pVIc complex the active site is in a groove; the four amino acids involved in catalysis are shown in purple[1,2]. The van der Waals spheres of pVIc are colored with the same color scheme as in Fig. 1d: basic amino acids are colored in bright red, hydrophobic in blue, polar in green, cysteine in yellow, and glycine in gray.

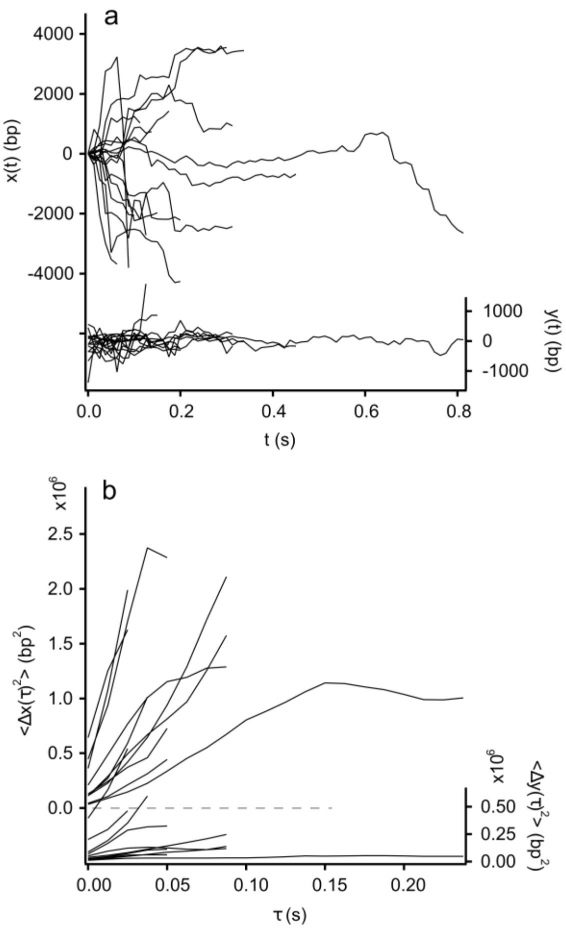

Supplementary Figure 2 l Diffusion of pVIc along flow-stretched dsDNA in 20 – 25 mM NaCl. (a) pVIc diffuses rapidly along DNA (x(t), left axis, 17 trajectories). (b) Mean-square displacement of 10 pVIc molecules shown in (a) sliding along DNA ($<\Delta x(t)^2>$, left axis). Motion transverse to the DNA (y(t) and $<\Delta y(\tau)^2>$, left axis), respectively, right axes) is represented on the same scale, as a control.

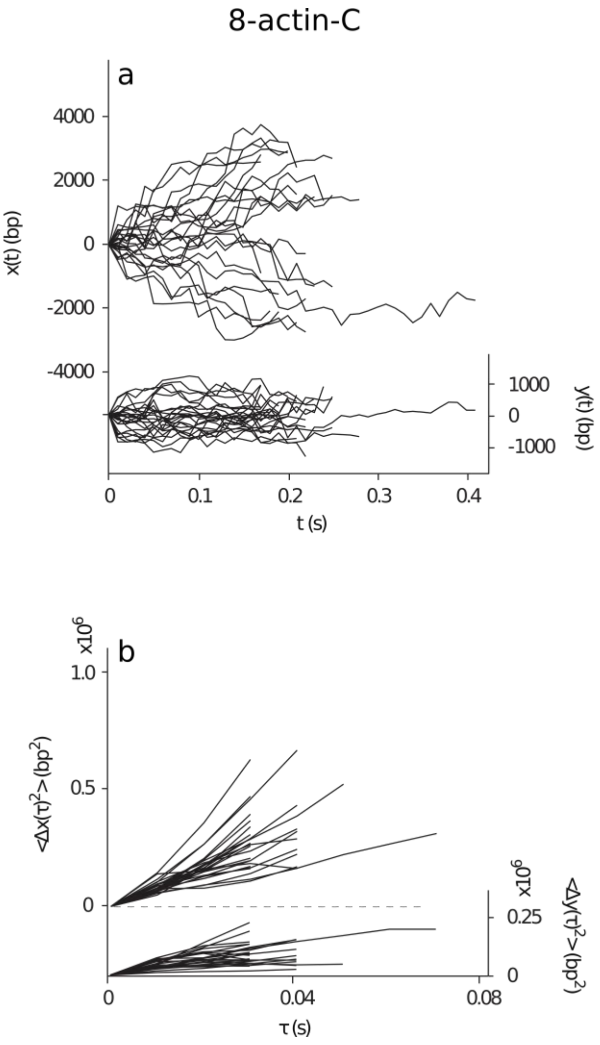

Supplementary Figure 3 l Diffusion of C-terminal peptides of β-actin along dsDNA. (a) The 8-amino acid peptide from the C-terminus of β-actin, 8-actin-C, diffuses-rapidly along DNA (69 trajectories). (b) Mean-square displacement of the trajectories shown in (a). (c) The 11-amino acid peptide from the C-terminus of β-actin, 11-actin-C, diffuses-rapidly along DNA (102 trajectories). (d) Mean-square displacement of the trajectories shown in (c). In a-d, motion transverse to the DNA, ($y(t)$ and $<\Delta y(\tau)^2>$, respectively, right axes) is represented on the same scale, as a control.

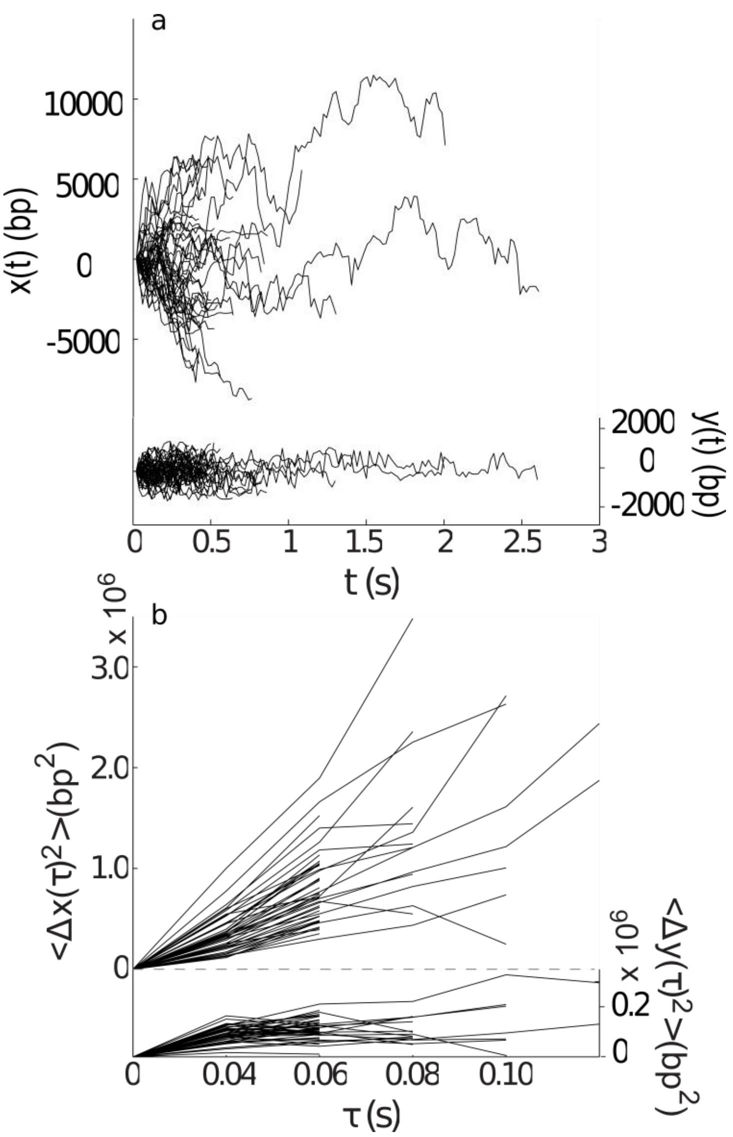

Supplementary Figure 4 l Diffusion of NLSIII peptide of the p53 protein along dsDNA. (a) The 13-amino acid peptide from the C-terminus of the p53 protein containing NLSIII, 13-p53-C (STSRHKKLMFKTE), diffuses-rapidly along DNA at pH 6.5 (45 trajectories). (b) Mean-square displacement of the trajectories shown in (a).

Supplementary Table 1

Initial reaction rate (**reaction half-time, $t_{1/2}$**)	**Reaction pathway**		
	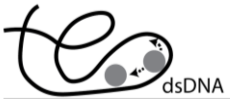	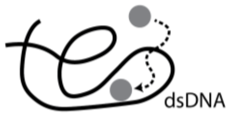	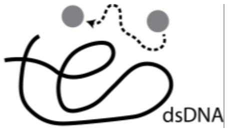
**Bimolecular association scenario**	**1D: on DNA**	**3D: solution to DNA**	**3D: in solution**
**All the molecules bind DNA & 1D diffusion occurs**	$k_A[A]_0^2$ **($t_{1/2}$ ≥ 6 - 60 s)**	k_A $([A]_0 K_D /[DNA])$ $[A]_0$ **($t_{1/2}$ ≥ 6600 s)**	k_A $([A]_0 K_D / [DNA])^2$ **($t_{1/2}$ ≥ 3 x 10^{10} s)**
**All the molecules bind DNA; no 1D diffusion**	no 1D rxn. on DNA	k_A $([A]_0 K_D /[DNA])$ $[A]_0$ **($t_{1/2}$ ≥ 6600 s)**	k_A $([A]_0 K_D / [DNA])^2$ **($t_{1/2}$ ≥ 3 x 10^{10} s)**
**One of the binding partners binds DNA**	no 1D rxn. on DNA	$(k_A/2)$ $[A]_0^2$ **($t_{1/2}$ ≥ 1 ms)**	k_A $([A]_0 / 2)^2$ **($t_{1/2}$ ≥ 2 ms)**
**No DNA binding**	no rxn. on DNA	no rxn. on DNA	k_A $[A]_0^2$ **($t_{1/2}$ ≥ 0.5 ms)**

Supplementary Table 1 I Bimolecular interactions on DNA and in solution. Abbreviations and Notes: Expressions for the initial reaction rates and approximate lower limits of time to react half the molecules in a population of molecules under different DNA-binding and 1D diffusion scenarios. $t_{1/2}$ values indicated are approximate lower limits and parameterized to correspond to the example in adenovirus maturation (see Supplementary Discussion for assumptions and parameter values). The dominant (smallest $t_{1/2}$) reaction pathway for each scenario can be identified by reading across the rows. $[A]_0$: initial concentration of each protein molecule type; K_D : equilibrium DNA-binding disassociation constant; k_A : bimolecular association rate constant; in 1D, $k_A = ((8D)(\pi t^{-1}))^{1/2}$ [3]; in 3D, $k_A = 4\pi\kappa D a$ [4].

Supplementary Note 1 | Binding of labeled pVIc to DNA

We know that AVP, pVI, AVP-pVIc complexes, and pVIc bind to DNA independent of DNA sequence. Their apparent equilibrium dissociation constants, $K_{d(app.)}$, are 63 nM[5], 46 nM[6], 4.6 nM[5], and 693 nM[7], respectively, Table 1. These numbers were determined by fluorescence anisotropy with the fluorophore attached to one of the 5'ends of dsDNA. However, to study other aspects of the interaction of pVIc with DNA, e.g. sliding, we needed to use pVIc with a fluorescent label attached to it. Accordingly, we labeled pVIc with the fluorescent dye Cy3B at Cys10'. Does the presence of this label on pVIc, as opposed to a label on DNA, affect the K_d for the binding of pVIc to DNA? The K_d was determined by fluorescence anisotropy. Aliquots of 12-mer dsDNA were added to 10 nM Cy3B labeled pVIc. When the changes in anisotropy were plotted versus the DNA concentration, a rectangular hyperbola was formed (Fig. 1a) signifying the $K_{d(app.)}$ was 264 ± 25 nM (Table 1). This is within a factor of 3 of the $K_{d(app.)}$ for the binding of pVIc to labeled DNA.

Supplementary Note 2 | Footprint of pVIc binding to DNA

We have previously measured the molecular footprint for the binding of pVI and AVP-pVIc complexes to DNA. One molecule of pVI covers 8 base pairs of dsDNA[8] and one molecule of an AVP-pVIc complex covers 6 base pairs of DNA[5], Table 1. However, we do not know the molecular footprint for the binding of pVIc to dsDNA. The stoichiometry of binding of labeled pVIc to DNA was ascertained using fluorescence anisotropy under "tight" binding conditions, conditions in which the concentration of one of the binding components is at least 10-fold greater than the $K_{d(app.)}$. Increasing amounts of 36-mer dsDNA were added to 10 µM pVIc (containing 10 nM Cy3B-labeled pVIc), and the change in anisotropy upon each addition was measured, Fig. 1b. Under these "tight" binding conditions at DNA concentrations below saturation of pVIc, all DNA present will be bound to pVIc. Above saturation, no added DNA will be able to bind to pVIc. As shown in Fig. 1b, as the concentration of DNA was increased, the anisotropy increased linearly. Once saturation was reached, there were no further increases in anisotropy as additional DNA was added. The data points could be characterized by two straight lines using a linear fitting routine. The intersection point of the two lines is the minimal concentration of DNA required to saturate 10 µM pVIc. Since this occurred at a concentration of 1700 nM 36-mer dsDNA, 10,000/1700 or 6 molecules of

labeled-pVIc bound to one molecule of 36-mer dsDNA. Similar experiments were done with 12-mer dsDNA and 18-mer dsDNA. One molecule of Cy3B-pVIc bound to one molecule of 12-mer dsDNA and 3 molecules of pVIc bound to one molecule of 18-mer dsDNA. When the number of molecules of pVIc bound to one DNA is plotted versus DNA length in base pairs, a straight line through the origin was observed, Fig. 1c. This implied that one molecule of pVIc occluded 7 base pairs of DNA, Table 1.

Supplementary Note 3 l Hopping and sliding on DNA

The mean one-dimensional diffusion constant for pVIc sliding on DNA was extremely large. Such a large diffusion constant caused us to consider the possibility that processes other than persistent ‘sliding’ along DNA might be occurring[9]. Sliding consists of one-dimensional translocation along the DNA with the protein in continuous contact with the DNA; it closely approximates idealized one-dimensional diffusion[9,10]. Hopping, also called microdiffusion, can under microscopic observation look like and be mistaken for one-dimensional diffusion[11,12]. Hopping occurs when the protein repeatedly dissociates from the DNA and rebinds at new locations on the DNA. In principle, hopping can lead to much faster apparent one-

dimensional translocation of a protein molecule along DNA than sliding, due to decreased friction of the protein with the solvent and the DNA[10].

Hopping and sliding by proteins with an electrostatic component to their binding free energies can be distinguished experimentally by measuring observed one-dimensional diffusion constants at different ionic strengths[13]. Were the observed protein motions occurring by hopping rather than sliding, increasing the salt concentration would decrease the residence time on DNA. This is because stronger electrostatic screening lowers the nonspecific binding affinity thereby increasing the fraction of time that the enzyme is unbound and mobile, and this gives rise to an apparent increase in the one-dimensional diffusion constant. For these reasons, the sliding activity of pVIc was assayed at two different salt concentrations, ~2 mM NaCl and ~20 mM NaCl. The data from the ~20 mM NaCl sliding assays are shown in Supplementary Figures 2a and b. $\langle D_1 \rangle$ was found to be $17.9 \pm 3.5 \times 10^6$ $(bp)^2$ s^{-1}, Table 1. Since this is not a higher value than the $26.0 \pm 1.8 \times 10^6$ $(bp)^2$ s^{-1} we observed in low salt, ~2 mM NaCl., Table 1, it appeared that the observed transport we observed in the sliding assays was dominated by sliding of pVIc in contact with the DNA and not by hopping.

Supplementary Note 4 l Rotational friction is rate limiting for AVP-pVIc sliding

Recently, we showed that molecules sliding along DNA, including AVP-pVIc complexes, diffuse along a helical path defined by DNA, rotating in order to keep the DNA-binding face of the protein in contact with DNA[10,14,15]. Based on this analysis, we estimate the free energy barriers to translocation average approximately 1 k_BT. That the peptide slides only slightly more rapidly than the AVP-pVIc complex (despite being much smaller and having lower friction with the solvent as a result), suggests that the peptide's configuration in the AVP-pVIc complex, Fig. 3 and Supplementary Fig. 1, is optimized by AVP to reduce free energy barriers to diffusion along DNA.

Supplementary Discussion

Many proteins with functions relevant to specific loci or features in the genome have been found capable of one-dimensional diffusion along DNA, an activity that can help to maintain biologically-relevant bimolecular association rates in the face of high concentrations of non-target DNA[16]. But what about proteins that must interact with one another in a similar

setting, e.g. inside a virus particle or the nucleus of a cell, with functions independent of specific loci or features on the genome? Would there be advantages to these proteins if they were to form bimolecular interactions via one-dimensional diffusion along DNA? Recently, we showed that the active form of the adenovirus proteinase and one of its substrates slide along DNA during activation and processing reactions, reactions that take place inside the virus particle but do not involve DNA metabolism[6,8,17,18].

The sliding assays were done under nonphysiological conditions to ensure accurate measurements of one-dimensional diffusion constants for sliding along DNA. The protein or peptide and DNA concentrations were low as was the ionic strength. The proteins or peptides were labeled with a fluorophore. To keep the fluorescence background low, the protein or peptide concentrations had to be kept low. The DNA concentration was well below the concentration it is in a virus particle. In the assay, the DNA is attached to a glass surface via a biotin-streptavidin linkage. High concentrations of DNA would result in their interaction thereby making sliding events difficult to interpret; for example jumping from one DNA strand to another. The ionic strength was kept low because increasing ionic strength decreases the association rate constant for a protein binding

to DNA[19,20]; it also decreases the residence time for a protein being bound to DNA (the time a protein slides along DNA)[13,21]. These conditions in the assay were required in order for there to be enough binding events to observe and quantitatively measure sliding without ambiguity.

Sequence-nonspecific DNA affinity can lead to widely disparate 3D (in solution) and 1D (on DNA) reactivities, creating an opportunity for the regulation of protein-protein interactions by targeting proteins to DNA, analogous to regulatory mechanisms that target interacting partners to membranes or molecular scaffolding[22,23]. Supplementary Table 1 shows equations for diffusion-limited initial reaction rates and approximate lower limit half-reaction times for protein-protein interactions for cases where all, half, or none of the molecules have DNA-binding activity. In the case corresponding to pVI and the adenovirus proteinase in virions (first row of Supplementary Table 1), the 1D reaction pathway dominates the 3D pathways by two to ten orders of magnitude. The potential for regulation by modulating sliding activity is apparent by comparing the first two rows of Supplementary Table 1 (the time for half the population to react is 100 - 1000 times slower in the absence of 1-D diffusion). The recruitment or

activation of a molecular sled is the first example of a general means to govern such a regulatory mechanism[6,17,18].

For simplicity, calculations for Supplementary Table 1 reflect the most basic case: diffusion-limited bimolecular self-reaction (A + A -> P, where P does not affect the activity of A). The initial rate expressions reflect the appropriate (1D or 3D) association rate constant k_A [3,4], the effect of differences in relative diffusion constants (1D diffusion constants are assumed as zero in determination of 3D relative diffusion constants), reduced 3D in-solution reactant concentration due to DNA-binding (through K_D and the DNA binding site concentration supposing that all the protein can simultaneously bind the DNA). In most of the indicated cases, the rates for alternative B + C -> P reactions with distinguishable binding partners are equal or modestly slower than A + A -> P when the concentrations of B and C each equal the concentration of A in the corresponding self-reaction case (see below for one exception).

$t_{1/2}$ is the diffusion-limited time required for half of the substrates to react. The values listed in Supplementary Table 1 are calculated using the below listed parameters approximating the diffusion-limited reaction of pVI

with the adenoviral proteinase inside virions and the reaction of the activated proteinase with other precursor proteins in the adenovirus virion.

virion interior volume	1.2×10^{-19}	liters
DNA length	3.5×10^{4}	bp $(\text{virion})^{-1}$
DNA length	1.1×10^{-5}	m $(\text{virion})^{-1}$
[DNA], binding site conc.	0.5	M
AVP & pVI K_D	5×10^{-8}	M
AVP & pVI D_{3D}	1×10^{-10}	$m^2\ s^{-1}$ (dilute solution)
AVP & pVI D_{3D}	1×10^{-11}	$m^2\ s^{-1}$ (in virion)
κ	0.001	unitless
AVP D_{1D}	2×10^{-12}	$m^2\ s^{-1}$
pVI D_{1D}	2×10^{-13}	$m^2\ s^{-1}$
AVP & pVI radius	1×10^{-9}	m
$[A]_0$	0.005	M

These parameters yield a realistic k_A for reaction in solution with no DNA: $3 \times 10^6\ M^{-1}\ s^{-1}$. The $t_{1/2}$ times for the 3D pathways when all the proteins have DNA-binding affinity are based on initial rates of reaction assuming the DNA binding equilibrium is fast with respect to the in-solution reaction.

An exception for the approximate equivalence of self-reaction (A + A -> P) and distinguishable interaction partners (B + C -> P) is the case for the 3D solution to solution reaction with one binding partner bound to DNA, where the B + C -> P rate will be significantly slower due to the low concentration of one binding partner in solution. Using parameters estimated for adenovirus, $t_{1/2}$ for this reaction is $\geq$ 3300 seconds when formulated as B + C -> P rather than self-reaction when using the initial rate of reaction presuming the DNA binding equilibrium is fast with respect to the in-solution reaction.

Supplementary References

1. McGrath, W.J., Ding, J., Sweet, R.M. & Mangel, W.F. Crystallographic structure at 1.6-Å resolution of the human adenovirus proteinase in a covalent complex with its 11-amino-acid peptide cofactor: insights on a new fold. *Biochem. Biophys. Acta* **1648**, 1-11 (2003).
2. Ding, J., McGrath, W.J., Sweet, R.M. & Mangel, W.F. Crystal structure of the human adenovirus proteinase with its 11 amino acid cofactor. *EMBO J.* **15**, 1778-1783 (1996).
3. Torney, D.C. & Mcconnell, H.M. Diffusion-limited reactions in one dimension. *The Journal of Physical Chemistry* **87**, 1941-1951 (1983).
4. Smoluchowski, M. v. Drei vortrage uber diffusion, brownsche bewegung und koagulation von kolloidteilchen. *Zeitschrift fur physik* **17**, 557-585 (1916).
5. McGrath, W.J. et al. Human adenovirus proteinase: DNA binding and stimulation of proteinase activity by DNA. *Biochemistry* **40**, 13237-13245 (2001).
6. Graziano, V. et al. Regulation of a viral proteinase by a peptide and DNA in one-dimensional space. II. Adenovirus proteinase is

activated in an unusual one-dimensional biochemical reaction. *J Biol Chem* **288**, 2068-2080 (2013).

7. Baniecki, M.L. et al. Interaction of the human adenovirus proteinase with its 11- amino-acid cofactor pVIc. *Biochemistry* **40**, 12349-12356 (2001).

8. Graziano, V. et al. Regulation of a viral proteinase by a peptide and DNA in one-dimensional space. I. Binding to DNA and to hexon of the precursor to protein VI, pVI, of human adenovirus. *J Biol Chem* **288**, 2059-2067 (2013).

9. von Hippel, P.H. & Berg, O.G. Facilitated target location in biological systems. *J. Biol. Chem.* **264**, 675-687 (1989).

10. Bagchi, B., Blainey, P.C. & Xie, X.S. Diffusion constant of a nonspecifically bound protein undergoing curvilinear motion along DNA. *J Phys Chem B* **112**, 6282-4 (2008).

11. Komazin-Meredith, G., Mirchev, R., Golan, D.E., van Oijen, A.M. & Coen, D.M. Hopping of a processivity factor on DNA revealed by single-molecule assays of diffusion. *Proc Natl Acad Sci U S A* **105**, 10721-6 (2008).

12. Gowers, D.M., Wilson, G.G. & Halford, S.E. Measurement of the contributions of 1D and 3D pathways to the translocation of a protein along DNA. *Proc. Natl. Acad. Sci. USA* **102**, 15883-15888 (2005).

13. Blainey, P.C., van Oijen, A.M., Banerjee, A., Verdine, G.L. & Xie, X.S. A base-excision DNA-repair protein finds intrahelical lesion bases by fast sliding in contact with DNA. *Proc Natl Acad Sci U S A* **103**, 5752-7 (2006).

14. Schurr, J. The one-dimensional diffusion coefficient of proteins absorbed on DNA. Hydrodynamic considerations. *Biophys. Chem.* **9**, 413-414 (1979).

15. Blainey, P.C. et al. Nonspecifically bound proteins spin while diffusing along DNA. *Nat Struct Mol Biol* **16**, 1224-9 (2009).

16. Berg, O.G. & von Hippel, P.H. Diffusion-controlled macro-molecular interactions. *Annu. Rev. Biophys. Chem.* **14**, 131-160 (1985).

17. Blainey, P.C. et al. Regulation of a viral proteinase by a peptide and DNA in one-dimensional space. IV. Viral proteinase slides along DNA to locate and process its substrates. *J Biol Chem* **288**, 2092-2102 (2013).

18. Baniecki, M.L., McGrath, W.J. & Mangel, W.F. Regulation of a viral proteinase by a peptide and DNA in one-dimensional space. III.

Atomic resolution structure of the nascent form of the adenovirus proteinase. *J Biol Chem* **288**, 2081-2091 (2013).

19. Barkley, M.D. Salt dependence of the kinetics of the lac repressor-operator interaction: role of nonoperator deoxyribonucleic acid in the association reaction. *Biochemistry* **20**, 3833-3842 (1981).

20. Winter, R.B., Berg, O.G. & von Hippel, P.H. Diffusion-driven mechanisms of protein translocation on nucleic acids. 3. The *Escherichia coli* lac repressor-operator interation: kinetic measurements and conclusions. *Biochemistry* **20**, 6961-6977 (1981).

21. Halford, S.E. An end to 40 years of mistakes in DNA-protein association kinetics. *Biochem. Soc. Trans.* **37**, 343-348 (2009).

22. Johnson, J.E. & Cornell, R.B. Amphitropic proteins: Regulation by reversible membrane interactions (review). . in *Molecular Membrane Biology*, Vol. 16 217-235 (1999).

23. Morrison, D. & R.J., D. Regulation of map kinase signaling modules by scaffold proteins in mammals. in *Annual Review of Cell and Developmental Biology*, Vol. 19 91-118 (2003).
